# Intrapulmonary Pharmacokinetics of First-line Anti-tuberculosis Drugs in Malawian Patients With Tuberculosis

**DOI:** 10.1093/cid/ciaa1265

**Published:** 2020-08-28

**Authors:** Andrew D McCallum, Henry E Pertinez, Laura J Else, Sujan Dilly-Penchala, Aaron P Chirambo, Irene Sheha, Madalitso Chasweka, Alex Chitani, Rose D Malamba, Jamilah Z Meghji, Stephen B Gordon, Geraint R Davies, Saye H Khoo, Derek J Sloan, Henry C Mwandumba

**Affiliations:** 1 Malawi-Liverpool-Wellcome Clinical Research Programme, University of Malawi College of Medicine, Blantyre, Malawi; 2 Department of Clinical Sciences, Liverpool School of Tropical Medicine, Liverpool, United Kingdom; 3 Institute of Translational Medicine, University of Liverpool, Liverpool, United Kingdom; 4 School of Medicine, University of St Andrews, St Andrews, United Kingdom

**Keywords:** tuberculosis, pulmonary, pharmacokinetics, pharmacodynamics, antitubercular

## Abstract

**Background:**

Further work is required to understand the intrapulmonary pharmacokinetics of first-line anti-tuberculosis drugs. This study aimed to describe the plasma and intrapulmonary pharmacokinetics of rifampicin, isoniazid, pyrazinamide, and ethambutol, and explore relationships with clinical treatment outcomes in patients with pulmonary tuberculosis.

**Methods:**

Malawian adults with a first presentation of microbiologically confirmed pulmonary tuberculosis received standard 6-month first-line therapy. Plasma and intrapulmonary samples were collected 8 and 16 weeks into treatment and drug concentrations measured in plasma, lung/airway epithelial lining fluid (ELF), and alveolar cells. Population pharmacokinetic modeling generated estimates of drug exposure (C_max_ and AUC) from individual-level post hoc Bayesian estimates of plasma and intrapulmonary pharmacokinetics.

**Results:**

One-hundred fifty-seven patients (58% HIV coinfected) participated. Despite standard weight-based dosing, peak plasma concentrations of first-line drugs were below therapeutic drug-monitoring targets. Rifampicin concentrations were low in all 3 compartments. Isoniazid, pyrazinamide, and ethambutol achieved higher concentrations in ELF and alveolar cells than plasma. Isoniazid and pyrazinamide concentrations were 14.6-fold (95% CI, 11.2–18.0-fold) and 49.8-fold (95% CI, 34.2–65.3-fold) higher in ELF than plasma, respectively. Ethambutol concentrations were highest in alveolar cells (alveolar cell–plasma ratio, 15.0; 95% CI, 11.4–18.6). Plasma or intrapulmonary pharmacokinetics did not predict clinical treatment response.

**Conclusions:**

We report differential drug concentrations between plasma and the lung. While plasma concentrations were below therapeutic monitoring targets, accumulation of drugs at the site of disease may explain the success of the first-line regimen. The low rifampicin concentrations observed in all compartments lend strong support for ongoing clinical trials of high-dose rifampicin regimens.

## Teaser

First-line anti-tuberculosis drugs achieve higher concentrations in the lung than in plasma in patients on treatment. Despite standard weight-based dosing, rifampicin concentrations are particularly low in plasma, epithelial lining fluid, and alveolar cells. Dose refinement may be required.

**(See the Editorial Commentary by Pasipanodya and Gumbo on pages e3374–6.)**

Although effective antibiotics for tuberculosis (TB) have been widely available for several decades, cure rates in high-burden countries remain variable [1]. Revisiting the pharmacokinetics-pharmacodynamics of first-line agents in patients with active pulmonary disease may identify opportunities to optimize or abbreviate anti-TB treatment.

Plasma pharmacokinetic indices for first-line anti-TB drugs vary by up to 10-fold between individuals [2–5] and their relationship with treatment response has varied across studies using differing methodologies [6–10]. We hypothesized that intrapulmonary pharmacokinetics will differ between individuals and may explain drug exposure–response relationships. Novel work using lung explants and spatial mass spectrometry has demonstrated gradients of both drug exposure and *Mycobacterium tuberculosis* drug sensitivity in granulomas and cavities [11, 12], but can only be performed in a small subset of patients with advanced disease.

In this study, drug concentrations in lung epithelial lining fluid and alveolar cells were used to assess intrapulmonary penetration, as expressed by epithelial lining fluid to plasma or alveolar cell to plasma concentration ratios. These ratios, described for a wide range of antimicrobials [13, 14], may inform an assessment of whether penetration is sufficient when related to the minimum inhibitory concentration (MIC) for the infecting organism [15, 16]. Few existing healthy volunteer–based data suggest extensive variability in the pulmonary penetration of first-line anti-TB drugs, with potentially subtherapeutic concentrations of rifampicin and isoniazid in epithelial lining fluid in a proportion of subjects [17–22]. These studies have limitations, however, as no patients with TB participated, drugs were given individually rather than in clinically relevant combinations, and sampling was limited to single fixed time points.

To address these limitations, we used bronchoalveolar lavage coupled with population pharmacokinetic modeling to assess site of disease pharmacokinetics in a cohort of adult patients with pulmonary TB receiving first-line anti-TB treatment.

## METHODS

### Study Design

This was a prospective, single-center, pharmacokinetic-pharmacodynamic study. Participants were sequentially assigned to 2 study arms—a plasma arm and an intrapulmonary arm—at a 2:1 allocation ratio.

The primary objective was to describe the pharmacokinetics (area under the concentration-time curve [AUC] and peak concentration [C_max_]) of rifampicin, isoniazid, pyrazinamide, and ethambutol in plasma, lung epithelial lining fluid, and alveolar cells. The secondary objective was to relate the pharmacokinetic indices to clinical treatment response.

### Study Participants

The study was conducted at Queen Elizabeth Central Hospital in Blantyre, Malawi, between January 2016 and October 2018. Patients registering for first-line treatment for TB at the hospital, or at health centers in urban Blantyre, were screened. Adults aged 16–65 years with sputum smear– or Xpert MTB/RIF-positive (Cepheid) pulmonary TB were eligible, while those with absolute or relative contraindications to research bronchoscopy were excluded [23] (Supplementary Text). Baseline chest radiographs were scored for severity of disease [24].

Participants received daily fixed-dose combination tablets according to a World Health Organization (WHO)–approved weight-adjusted regimen and national guidelines [25]. All participants had human immunodeficiency virus (HIV) antibody tests and those who tested positive received antiretroviral therapy [26]. Participants were followed up for 18 months. Adherence was monitored by direct questioning and pill counts.

### Pharmacokinetic Sampling

Pharmacokinetic sampling was completed at 7–8 and 15– 16 weeks into TB treatment. All participants were observed to take their anti-TB treatment after an overnight fast. Those in the plasma arm had blood samples collected at 1 and 3 or 2 and 4 hours postdose. Those in the intrapulmonary arm had blood samples collected predose and at 0.5, 1, 3, and 5 hours or 2, 4, 6, and 8 hours postdose.

Participants in the intrapulmonary arm had a research bronchoscopy at 2, 4, or 6 hours postdose. Bronchoscopy was performed as previously described [23], with lavage of the right middle lobe. A cell count was obtained by hemocytometry, before centrifugation to obtain samples of cell pellets and supernatant. The volume of epithelial lining fluid in bronchoalveolar lavage was calculated using the urea dilution method [13, 17–20].

Rifampicin, isoniazid, pyrazinamide, and ethambutol concentrations were measured in plasma, bronchoalveolar lavage supernatant, and cell pellets with a 4-drug liquid chromatography–tandem mass spectrometry assay using appropriate internal standards validated to internationally recognized acceptance criteria (Supplementary Text).

### Pharmacokinetic Modeling

Population pharmacokinetic models were developed using NONMEM (version 7.4.0; ICON Development Solutions). A 2-stage model-building strategy was used: first, the plasma-only pharmacokinetic models were developed for each drug; then the plasma and intrapulmonary pharmacokinetic data were analyzed under a combined model (Supplementary Text).

One- and two-compartment pharmacokinetic disposition models with alternative models of absorption were explored in fittings to the plasma data. Base model selection was achieved using likelihood ratio testing with the minimum objective function value as the criterion, and examination of relative standard error values and goodness-of-fit plots. Stepwise generalized additive modeling was then used to identify significant covariates for the model (from among age, weight, body mass index, sex, HIV status, and creatinine clearance). The plasma models were evaluated using visual predictive checks.

Concentrations in the epithelial lining fluid and alveolar cell compartments were characterized by ratio parameters relative to their plasma concentration (*R*_ELF_ and *R*_AC_, respectively). Interindividual variability in *R*_ELF_ and *R*_AC_ was incorporated using an exponential error model with separate residual error variances estimated for both epithelial lining fluid and alveolar cells. *R*_ELF_ is based on total drug (protein-unbound plus bound), whereas only the unbound fraction is pharmacologically active and able to be transported or diffused into the epithelial lining fluid [27]. Of the 4 first-line drugs, only rifampicin has extensive protein binding. Assuming an unbound fraction of rifampicin in plasma of 20% [27], and negligible protein binding in epithelial lining fluid [28], we approximated the *R*_ELF/unbound-plasma_ ratio to be 5-fold greater than *R*_ELF/total-plasma_.

Model-based estimates of individual values of AUC, T_max_ (time to peak concentration), and C_max_ were calculated from empirical Bayes estimates of parameters (Supplementary Text). Plasma C_max_ estimates were compared with therapeutic drug-monitoring targets: rifampicin ≥8 μg/mL, isoniazid ≥3 μg/mL, pyrazinamide ≥20 μg/mL, and ethambutol ≥2 μg/mL [29]. Rifampicin AUC >13 μg · hour/mL, isoniazid AUC >52 μg · hour/mL, pyrazinamide C_max_ >35 μg/mL, and AUC >363 μg · hour/mL were also considered given associations with improved outcomes in 1 analysis [7, 30].

### Treatment Response and Bacteriology

Sputum samples were collected at treatment initiation, 2 months, and at end-of-treatment. Baseline drug susceptibility of screening isolates was measured on custom-made microtiter plates (UKMYC3 Sensititre; Thermo Scientific). These assays used 96-well plates with increasing concentrations of rifampicin (0.015–16 µg/mL), isoniazid (0.015–16 µg/mL), and ethambutol (0.25–16 µg/mL) (Supplementary Text).

Two-month culture conversion in liquid media was captured. Participants with negative TB sputum cultures from end-of-treatment onwards or who stopped coughing and remained well after treatment were defined as having a favorable clinical outcome. Recurrent TB (relapse/reinfection), failed treatment, and death were grouped together as unfavorable clinical outcomes. Logistic regression, chi-square test, and Fisher’s exact test were used to assess relationships between pharmacokinetics and treatment response.

### Ethics

Ethical approval was obtained from the Research Ethics Committees of the College of Medicine, University of Malawi, and the Liverpool School of Tropical Medicine.

## RESULTS

### Demographic and Clinical Description of Cohort

We recruited 157 adult patients with pulmonary TB (Table 1). The median age was 34 years (interquartile range [IQR], 28–39 years), 76.4% were male (120/157), and 58.0% had HIV coinfection (91/157). More than half of coinfected participants (57.1%, 52/91) were diagnosed with HIV during TB diagnosis.

**Table 1.** **Demographic and Clinical Description of the Cohort**

Characteristics	Total (N = 157)	Intrapulmonary Arm (n = 51)	Plasma Arm (n = 106)
Age, median [IQR], years	34 [28–39]	32 [26–36]	34 [28–41]
Male sex, n (%)	120 (76.4)	44 (86.3)	76 (71.7)
Weight, median [IQR], kg	51.1 [46.9–55.6]	50.0 [47.3–53.8]	52.0 [46.6–56.5]
Body mass index, median [IQR], kg/m^2^	18.4 [17.0–19.8]	17.9 [16.8–18.9]	18.7 [17.1–20.1]
HIV coinfection, n (%)	91 (58.0)	23 (45.1)	68 (64.2)
Baseline CD4 count in patients with HIV, median [IQR], cells/mm^3^	178 [80–285]	178 [93–273]	175 [77–284]
On antiretroviral therapy at baseline if coinfection with HIV, n (%)	39 (42.9)	10 (43.5)	29 (42.6)
Rifampicin/isoniazid/pyrazinamide/ethambutol dose, n (%)			
300/150/800/550 mg	3 (1.9)	1 (2.0)	2 (1.9)
450/225/1200/825 mg	107 (68.2)	38 (74.5)	69 (65.1)
600/300/1600/1100 mg	45 (28.7)	12 (23.5)	33 (31.1)
750/375/2000/1375 mg	2 (1.3)	0 (0.0)	2 (1.9)
Adherence,^a^ n (%)			
Missed no doses	135 (86.0)	41 (80.4)	94 (88.7)
Missed 1–2 doses	14 (8.9)	6 (11.8)	8 (7.5)
Missed >2 doses	8 (5.1)	4 (7.8)	4 (3.8)

Abbreviations: HIV, human immunodeficiency virus; IQR, interquartile range.

^a^Adherence assessed by direct questioning and pill counts.

The use of fixed-dose combination regimens resulted in administration of a median rifampicin dose of 9.4 mg/kg (IQR, 8.8–10.3), isoniazid of 4.7 mg/kg (IQR, 4.4–5.1), pyrazinamide of 25.2 mg/kg (IQR, 23.5–27.4), and ethambutol of 17.3 mg/kg (IQR, 16.2–18.8). Four (2.5%) participants were underdosed for rifampicin and isoniazid, and 5 (3.2%) for ethambutol according to the WHO-recommended range (rifampicin, 10 mg/kg; isoniazid, 5 mg/kg; pyrazinamide, 25 mg/kg; ethambutol, 15 mg/kg) [31]. No correlation was seen between the radiological extent of right midzone disease and intrapulmonary drug concentration (Supplementary Figure 1).

### Rifampicin Pharmacokinetics

The rifampicin dataset comprised 741 plasma concentration-time observations from 140 participants. The plasma data were best described by a 1-compartment disposition model with first-order absorption and elimination. Only sex and HIV status were identified as significant covariates: the final model showed 32% greater clearance in male participants (CL/F; 16.1 L/hour in males vs 12.2 L/hour in females), and 34% greater volume of distribution in participants with HIV (V/F; 29.7 L in those with HIV vs 22.2 L in those without HIV). The visual predictive check and goodness-of-fit plots indicated that the final plasma model performed adequately (Supplementary Figures 2 and 3).

Rifampicin concentrations in epithelial lining fluid and alveolar cells exceeded those in plasma. Eighty-three sparse concentration-time observations in epithelial lining fluid and 89 in alveolar cells, from 51 participants, were included in the final combined intrapulmonary model. The final model parameters are included in Supplementary Table 1. The extent of distribution to epithelial lining fluid was near double that of plasma with a typical predicted *R*_ELF_ of 1.97 (95% confidence interval [CI], 1.65–2.30) and was also elevated for alveolar cells (*R*_AC_, 1.35; 95% CI, .89–1.81). After accounting for plasma protein binding, the *R*_ELF/unbound-plasma_ ratio will be approximately 5-fold greater than the *R*_ELF/total-plasma_ recorded here: 9.85 compared to 1.97.

**Figure 1.**
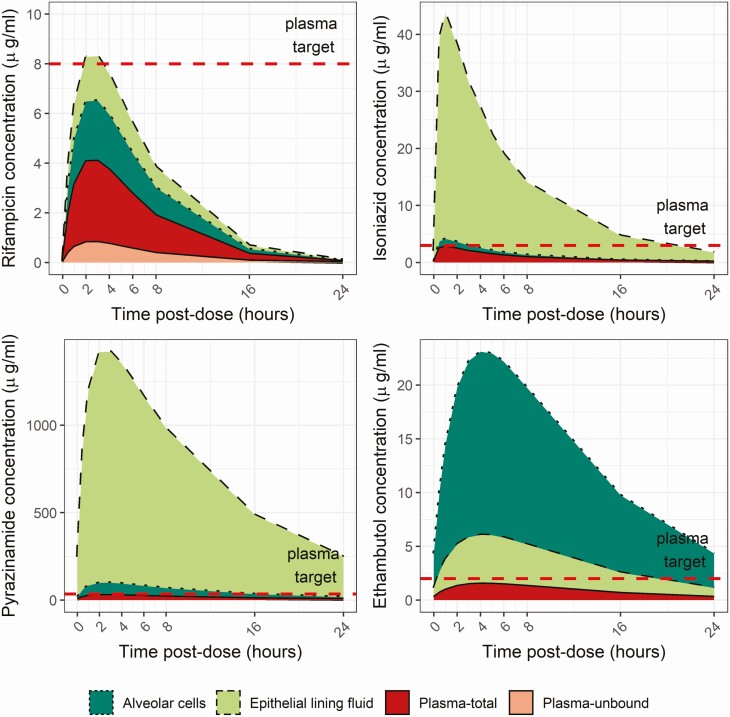
Summary concentration-time plots for rifampicin, isoniazid, pyrazinamide, and ethambutol in plasma, epithelial lining fluid, and alveolar cells from population means at steady state. To account for rifampicin protein binding, the red/pink-shaded area in the top-left panel illustrates plasma drug exposure for total drug (top line) or unbound drug (bottom line), assuming 80% protein binding in plasma and negligible protein binding in epithelial lining fluid. Plasma concentrations for isoniazid, pyrazinamide, and ethambutol are shown as total drug only. Concentrations at the different time points were calculated using the Bayesian posterior pharmacokinetic parameter value estimates and epithelial lining fluid to plasma (*R*_ELF_) and alveolar cells to plasma (*R*_AC_) ratios. The horizontal dotted line represents the plasma targets for therapeutic drug monitoring [29].

Rifampicin drug exposure in plasma was low relative to therapeutic drug-monitoring targets (C_max_, 4.0 μg/mL [3.5–4.8] for total drug; target ≥8 μg/mL [29]) (Figure 1 and Table 2). Rifampicin drug exposure was greatest in the epithelial lining fluid compared with plasma and alveolar cells.

**Table 2.** **Final Steady-state Parameter Estimates for Drug Exposure (AUC and C**_**max**_**) in Plasma, Epithelial Lining Fluid, and Alveolar Cells**

Drug and Pharmacokinetic Index	Plasma Therapeutic Drug-monitoring Target [29]	Matrix, Median (IQR)		
		Plasma	Epithelial Lining Fluid	Alveolar Cells
Rifampicin				
AUC, μg · h/mL	…	6.6 [5.6–7.5]^a^	65.7 [52.4–77.1]	46.0 [36.4–55.1]
AUC. μg · h/mL	…	30.6 [27.3–36.7]^b^	…	…
C_max_, μg/mL	…	0.8 [0.3–1.0]^a^	7.8 [6.4–10.0]	5.3 [4.4–7.6]
C_max_, μg/mL	≥8	4.0 [3.5–4.8]^b^	…	…
Isoniazid				
AUC, μg · h/mL	…	19.0 [11.9–25.9]	277.8 [154.9–382.4]	24.4 [14.1–34.8]
C_max_, μg/mL	≥3	2.6 [2.4–3.0]	38.9 [32.8–46.2]	3.5 [2.9–4.2]
Pyrazinamide				
AUC, μg · h/mL	…	319.5 [292.0–384.3]	16 062 [14 096–19 403]	1036 [899–1272]
C_max_, μg/mL	≥20^c^	24.0 [22.2–26.3]	1195 [1062–1361]	78 [68–94]
Ethambutol				
AUC, μg · h/mL	…	5.4 [2.9–7.4]	22.6 [11.7–29.2]	83.0 [48.0–110.1]
C_max_, μg/mL	≥2	1.3 [1.1–1.6]	5.2 [4.2– 6.6]	20.0 [15.4–25.3]

Abbreviations: AUC, area under the concentration-time curve; C_max_, peak concentration; h, hour; IQR, interquartile range.

^a^Estimated unbound fraction of rifampicin in plasma, assuming typical 80% rifampicin protein binding.

^b^Total rifampicin in plasma.

^c^Pyrazinamide C_max_ ≥35 μg/mL has also been proposed as a target due to association with improved outcomes [30].

### Isoniazid Pharmacokinetics

The isoniazid plasma dataset contained 750 concentration-time observations from 140 participants, best described by a 1-compartment pharmacokinetic model with first-order absorption and elimination. Two-compartment models were met with some improvement in the objective function value but unacceptably poor precision of parameter estimates. Weight as an effect on volume of distribution (V/F) was the only covariate that significantly improved the model fit, with the volume of distribution increasing by 1.08 L for every kilogram above the population mean of 51.1 kg.

Eighty-eight epithelial lining fluid and 89 alveolar cell concentration-time observations from 51 participants were available for the second stage of modeling. Isoniazid distributed extensively to epithelial lining fluid, with concentrations nearly 15-fold greater than plasma (*R*_ELF_, 14.6; 95% CI, 11.2–18.0) (Supplementary Table 1). Concentrations in alveolar cells were closer to plasma (*R*_AC_, 1.31; 95% CI, .95–1.67). The high concentrations achieved in epithelial lining fluid are demonstrated on the summary concentration-time plot (Figure 1) and estimates in Table 2.

### Pyrazinamide Pharmacokinetics

A total of 409 observations from 131 participants were modeled. Inclusion of weight as a covariate for both clearance (CL/F) and volume of distribution (V/F) significantly improved the model fit.

Fifty concentration-time observations were available for intrapulmonary pharmacokinetic modeling (Supplementary Table 1). Pyrazinamide was extensively distributed to epithelial lining fluid, achieving an *R*_ELF_ of 49.8 (95% CI, 34.2–65.3). Median pyrazinamide C_max_ in epithelial lining fluid was 1195 μg/mL (IQR, 1062–1361), as compared with 24.0 μg/mL (IQR, 22.2–26.3) in plasma (Table 2). Pyrazinamide was observed to accumulate in alveolar cells, with concentrations 3.18-fold greater than in plasma (95% CI, 1.90–4.46). No patients achieved the proposed C_max_ target of >35 μg/mL, while only 37 (23.1%) patients achieved an AUC >363 μg · hour/mL.

### Ethambutol Pharmacokinetics

A total of 416 ethambutol plasma concentration-time observations were modeled. Again, a 1-compartment disposition model with first-order absorption was sufficient to describe the data and attempts to use a 2-compartment system or incorporate an absorption lag phase were met with nonconvergence. After stepwise backwards elimination, only creatinine clearance as an effect on clearance (CL/F) was retained as a covariate in the model.

Fifty epithelial lining fluid and alveolar cell concentration-time observations for ethambutol were available. Final parameter estimates from the final intrapulmonary pharmacokinetic model showed that, in contrast to the other 3 first-line drugs, the highest ethambutol levels were seen in the alveolar cells (*R*_AC_, 15.0; 95% CI, 11.4–18.6) (Supplementary Table 1 and Figure 1), while concentrations in epithelial lining fluid were 4-fold higher than in plasma (95% CI, 3.3–4.6-fold). While median plasma C_max_ was low at 1.3 μg/mL (IQR, 1.1–1.6), higher peak concentrations of 5.2 μg/mL (IQR, 4.2–6.6) and 20.0 μg/mL (15.4–25.3) were seen in epithelial lining fluid and alveolar cells, respectively.

### Drug Susceptibility

**Figure 2.**
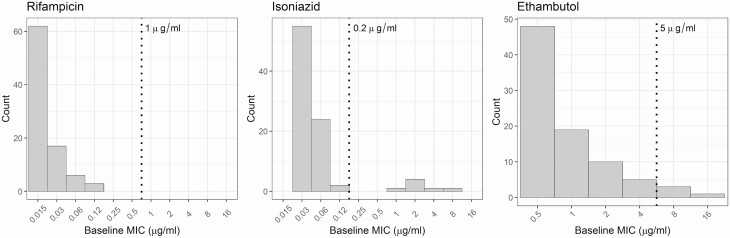
Baseline drug sensitivity. Drug sensitivity in baseline sputum *Mycobacterium tuberculosis* isolates were determined using microtiter plates (n = 88). Pyrazinamide was not assessed due to its need for acidic test conditions. The MIC was recorded as the lowest concentration in the microtiter plate with no visible growth observed. The currently recommended critical concentration (breakpoint) is indicated by the vertical dashed line [32]. Abbreviation: MIC, minimum inhibitory concentration.

Baseline isolates (n = 88) were highly sensitive to rifampicin: the modal MIC was 0.015, 0.03, and 0.5 μg/mL for rifampicin, isoniazid, and ethambutol, respectively (Figure 2). A total of 100 000 AUC, C_max_, and MIC values were generated from the mean and standard deviation using Monte-Carlo simulation, and used to describe the likely distribution of AUC/MIC and C_max_/MIC in this cohort (Table 3).

**Table 3.** **Summary of Pharmacokinetic Indices from Modeled Plasma Data**

Drug	Simulated Plasma Median AUC/MIC [IQR]	Simulated Plasma Median C_max_/MIC [IQR]
Rifampicin^a^	2319 [1422–3786]	285.0 [178.3–456.2]
Isoniazid	448 [438–458]	75.4 [66.7–85.3]
Pyrazinamide^b^	…	…
Ethambutol	64 [17–239]	4.0 [1.1–14.6]

MIC data were available from baseline isolates from 88 participants. Using the mean and standard deviation for AUC, C_max_, and MIC from this dataset, 100 000 AUC/MIC and C_max_/MIC pairings were generated using Monte-Carlo simulation to estimate the data distribution.

Abbreviations: AUC, area under the concentration-time curve; C_max_, peak concentration; IQR, interquartile range; MIC, minimum inhibitory concentration.

^a^Rifampicin AUC and C_max_ based on total (protein bound + unbound) drug.

^b^MIC data not available for pyrazinamide.

### Relationship With Clinical Outcome

A total of 126 participants had sufficient data to assess 2-month culture conversion: 81 (64%) had stable culture conversion by 2 months. Clinical outcome was captured in 133 participants, with 15 (11%) unfavorable outcomes recorded. Two-month culture conversion was not predictive of clinical outcome (*P* < .05, McNemar’s hypothesis test for paired data). We observed no relationship between rifampicin AUC >13 μg · hour/mL, isoniazid AUC >52 μg · hour/mL, or pyrazinamide AUC >363 μg · hour/mL and clinical outcome (*P* = 1.000, Fisher’s exact test), nor any association between baseline MIC and 2-month culture conversion or final outcome by logistic regression. None of the plasma or intrapulmonary pharmacokinetic parameters were associated with culture conversion or late clinical outcome (Table 4).

**Table 4.** **Pharmacokinetic Indices and Treatment Response**

Pharmacokinetic Parameter			OR for 2-Month Culture Conversion (95% CI)	*P*	OR for Favorable Outcome (95% CI)	*P*
Drug	Matrix	Pharmacokinetic Index				
Rifampicin	Plasma	AUC (μg · h/mL)	1.01 (.95–1.07)	.815	1.03 (.95–1.14)	.488
		C_max_ (μg/mL)	1.02 (.73–1.44)	.918	1.68 (.91–3.47)	.125
	Epithelial lining fluid^a^	AUC (μg · h/mL)	1.02 (1.00–1.04)	.118	1.09 (.99–1.43)	.321
		C_max_ (μg/mL)	1.17 (1.01–1.45)	.085	2.03 (.97–9.24)	.221
	Alveolar cells^a^	AUC (μg · h/mL)	1.00 (.99–1.02)	.607	1.00 (.99–1.05)	.836
		C_max_ (μg/mL)	1.05 (.93–1.19)	.429	1.03 (.89–1.43)	.801
Isoniazid	Plasma	AUC (μg · h/mL)	.98 (.94–1.02)	.281	1.07 (.99–1.18)	.100
		C_max_ (μg/mL)	1.24 (.58–2.68)	.583	3.79 (.96–17.86)	.071
	Epithelial lining fluid^a^	AUC (μg · h/mL)	1.00 (1.00–1.00)	.372	1.00 (1.00–1.01)	.575
		C_max_ (μg/mL)	1.02 (1.00–1.04)	.116	1.03 (.98–1.15)	.400
	Alveolar cells^a^	AUC (μg · h/mL)	1.00 (.98–1.02)	.992	1.00 (.98–1.06)	.980
		C_max_ (μg/mL)	1.04 (.85–1.30)	.674	1.01 (.73–1.77)	.959
Pyrazinamide	Plasma	AUC (μg · h/mL)	1.00 (.99–1.00)	.303	1.00 (1.00–1.01)	.751
		C_max_ (μg/mL)	1.01 (.91–1.13)	.869	1.07 (.90–1.29)	.464
	Epithelial lining fluid^a^	AUC (μg · h/mL)	1.00 (1.00–1.00)	.652	1.00 (1.00–1.00)	.356
		C_max_ (μg/mL)	1.00 (1.00–1.00)	.217	1.00 (1.00–1.01)	.341
	Alveolar cells^a^	AUC (μg · h/mL)	1.00 (1.00–1.00)	.276	1.00 (1.00–1.00)	.443
		C_max_ (μg/mL)	1.00 (.99–1.01)	.483	1.01 (.99–1.06)	.489
Ethambutol	Plasma	AUC (μg · h/mL)	1.08 (.92–1.27)	.360	.91 (.71–1.17)	.465
		C_max_ (μg/mL)	.85 (.32–2.29)	.743	1.12 (.25–5.86)	.889
	Epithelial lining fluid^a^	AUC (μg · h/mL)	0.97 (.82–1.12)	.658	1.06 (.89–1.73)	.733
		C_max_ (μg/mL)	0.98 (.70–1.35)	.878	1.05 (.71–2.32)	.856
	Alveolar cells^a^	AUC (μg · h/mL)	0.99 (.95–1.02)	.469	1.07 (.97–1.28)	.345
		C_max_ (μg/mL)	0.98 (.91–1.05)	.584	1.11 (.92–1.55)	.420

Abbreviations: AUC, area under the concentration-time curve; CI, confidence interval; C_max_, peak concentration; h, hour; OR, odds ratio.

^a^Analysis restricted to those in the intrapulmonary group.

## DISCUSSION

We observed low plasma drug concentrations for all 4 first-line anti-TB drugs in Malawian adults, relative to established therapeutic drug-monitoring targets [29]. In addition, we report differences in drug concentrations between plasma and pulmonary compartments. Rifampicin concentrations were low in both compartments, suggesting that the current recommended dose is not optimal. While plasma concentrations of isoniazid, pyrazinamide, and ethambutol were also low, these drugs achieved higher concentrations in epithelial lining fluid and alveolar cells; accumulation at the tissue site of disease may be important for the success of first-line anti-TB therapy.

The observed low plasma drug concentrations were consistent with other studies of steady-state pharmacokinetics from patients with TB in high-endemicity settings [2, 33–36]. Target concentrations for therapeutic drug monitoring were derived from descriptions of drug exposure in both patients and healthy volunteers, and have been associated with clinical outcomes in some studies [10, 29]. Furthermore, most patients in our study did not achieve the proposed C_max_ (pyrazinamide: 35 μg/mL) or AUC cutoffs (isoniazid: 52 μg · hour/mL; pyrazinamide: 363 μg· hour/mL) identified as predictive of long-term response [7]. Taken together, we suggest that current dosing might be too low to achieve pharmacokinetic targets.

All 4 drugs achieved higher concentrations in epithelial lining fluid and alveolar cells than plasma, with isoniazid and pyrazinamide 15- and 50-fold higher in lining fluid than plasma, respectively, and ethambutol concentrating within the cells. Our findings differ from previous work, where rifampicin epithelial lining fluid concentrations were reported to be one-fifth of plasma concentrations [17], and less extensive distribution of isoniazid and pyrazinamide to lining fluid was observed [18, 19]. Whereas previous work occurred in healthy volunteers (with or without HIV), the samples in the present study came from a cohort of adult patients with active pulmonary TB, many of whom were significantly immunosuppressed. Active inflammation, with increased permeability, cellular influx, and disruption of the blood–alveolar barrier may alter tissue drug penetration in those with ongoing infection [27]. Peak intrapulmonary concentrations were seen 2 hours after drug administration, suggesting that single pharmacokinetic measurements at 4 hours in previous studies underestimated drug exposure.

Despite low plasma drug concentrations, 89% of our cohort had a favorable clinical outcome. The AUC/MIC and C_max_/MIC for first-line drugs have been described as drivers of treatment efficacy in preclinical models [37–40], but local variation in MICs of non–genotypically resistant *M. tuberculosis* isolates against first-line TB drugs has not been well described. High plasma and intrapulmonary AUC/MIC and C_max_/MIC ratios [8, 39] were achieved in this cohort as a result of steady-state accumulation of drugs at the site of disease, coupled with preserved drug sensitivity, particularly for rifampicin.

Dose optimization may yield further improvements in treatment outcomes. Only two-thirds (64%) of the patients had culture converted by 2 months, despite drug-sensitive disease, good adherence, and intensive follow-up. We postulate that the slow bacillary clearance and unfavorable clinical outcomes are a consequence of the observed plasma and intrapulmonary rifampicin exposure. Rifampicin-dosing studies have demonstrated that increased dosing may be associated with supra-proportional increases in plasma AUC [41] and improved early bactericidal activity [42]. These studies have typically used milligram/kilogram doses several-fold greater than used in the present study, which may markedly increase rifampicin exposure within the intrapulmonary compartment. Participants with HIV were observed to have a greater volume of distribution for rifampicin, likely reflecting reduced bioavailability in coinfected patients [43].

We were unable to detect a relationship between plasma or intrapulmonary pharmacokinetics and treatment response in this cohort using standard approaches. Exploratory methods such as those based on machine learning might offer further insights into these relationships and may form the basis of future work. Given a relatively narrow exposure across a constant milligram/kilogram range using weight-banded dosing, studies assessing a greater range of dosing may be required to fully delineate relationships between pharmacokinetics and binary clinical outcomes. Alternatively, modeled bacillary elimination rates, as a continuous measure of treatment response, may identify pharmacokinetic-pharmacodynamic relationships from studies of fewer participants [42, 44]. Even if dose refinement is not associated with improved rates of relapse-free cure, identification of factors associated with more rapid bacillary elimination may be important for interruption of transmission and reduction of post-TB lung disease.

There were several limitations to our study. Bronchoalveolar lavage may result in drug efflux from macrophages, but these effects were minimized by placing samples on ice and rapid centrifugation. The cell pellet obtained from bronchoalveolar lavage contains a mixture of alveolar macrophages, epithelial cells, lymphocytes, and other leukocytes, and the drug concentration is recorded in micrograms per milliliter of cells. Alveolar macrophages represent more than 80% of cells in lavage, and are the largest cells retrieved [23], and as such, the alveolar cell concentrations are more likely to be an underestimation of macrophage drug concentration. Finally, we were only able to measure MICs from a subset of baseline isolates, instead using summary estimates to describe AUC/MIC and C_max_/MIC distributions for the cohort.

These are the only data describing the steady-state intrapulmonary pharmacokinetics of first-line anti-TB therapy in patients with pulmonary disease. Ultimately, understanding the mismatch in antibiotic exposure between plasma and site of infection may aid dose refinement and development of optimal pan-TB regimens for both drug-sensitive and drug-resistant disease.

## Supplementary Data

Supplementary materials are available at *Clinical Infectious Diseases* online. Consisting of data provided by the authors to benefit the reader, the posted materials are not copyedited and are the sole responsibility of the authors, so questions or comments should be addressed to the corresponding author.

ciaa1265_suppl_Supplementary_Materials

## Notes

***Acknowledgments.*** The authors thank the staff of the Clinical Investigation Unit, Ward 3A, and Radiology Department, Queen Elizabeth Central Hospital; the TB Officers of the Blantyre District Health Office and the Malawi-Liverpool-Wellcome Clinical Research Programme for their support and cooperation during the study. We are grateful to Jehan Ghany and Elizabeth Joekes for their work on the chest radiograph scoring. Lisa Stone, Liverpool School of Tropical Medicine, was integral to some of the laboratory work described in this manuscript but sadly passed away before it was completed; we gratefully acknowledge her contribution. Finally, we thank all study participants for their enthusiastic participation in the study, without whom this work would not be possible.

***Financial support.*** This work was supported by a Wellcome Trust Clinical PhD Fellowship (grant number 105392/B/14/Z; to A. D. M.). The Malawi-Liverpool-Wellcome Clinical Research Programme is supported by a strategic award from the Wellcome Trust. We also acknowledge infrastructural support for bioanalysis from the Liverpool Biomedical Research Centre funded by Liverpool Health Partners.

***Potential conflicts of interest.*** The authors: No reported conflicts of interest. All authors have submitted the ICMJE Form for Disclosure of Potential Conflicts of Interest.
