# Supplementary material for: Intrapulmonary Pharmacokinetics of First-line Anti-tuberculosis Drugs in Malawian Patients With Tuberculosis
Source: Clin Infect Dis. 2020 Aug 28;73(9):e3365–73. doi: 10.1093/cid/ciaa1265 (PMC8563277; doi:10.1093/cid/ciaa1265)
Supplement: ciaa1265_suppl_Supplementary_Materials [file ciaa1265_suppl_supplementary_materials.docx]

**Supplementary text**

*Exclusion criteria*

Exclusion criteria included haemoglobin <8 g/dl, creatinine level >177 μmol/l (2 mg/dl), total bilirubin >51 μmol/l (3 mg/dl), alanine transaminase level >200 IU/l, clinical status suggestive of imminent mortality (World Health Organization Performance Score 4) [1], pregnancy, tuberculosis treatment within 5 years, or corticosteroid therapy. Participants with microbiologically-unconfirmed TB (no positive culture or Xpert MTB/RIF result) were excluded.

*Bioanalytical methods*

Samples of plasma, epithelial lining fluid (ELF), and alveolar cells (AC) were transported on dry ice to the Bioanalytical Facility, University of Liverpool, for batch analysis. Rifampicin, isoniazid, pyrazinamide and ethambutol concentrations were measured using a simultaneous four-drug liquid chromatography / tandem mass spectrometry assay using appropriate stable isotope-labelled internal standards validated to FDA acceptance criteria. The lower limits of quantification for the assays were 15, 20, 200 and 7 ng/ml for rifampicin, isoniazid, pyrazinamide, and ethambutol, respectively.

Stock solutions containing 1 mg/ml rifampicin, 5 mg/ml isoniazid, 10 mg/ml pyrazinamide, and 5 mg/ml ethambutol (Sigma Aldrich) were prepared in methanol. A working internal standard solution containing 200 mg/ml rifampicin-d3, 500 ng/ml isoniazid-d4, 1 µg/ml pyrazinamide-15N,d3, and 200 ng/ml ethambutol-d4-dihydrochloride (Toronto Research Chemicals) was made up in methanol.

Serial drug dilutions in plasma were used to generate standard curves for peak area ratios of drug/internal standard on the chromatogram over an appropriate concentration range. Concentrations of TB drugs from clinical samples were calculated from their peak area ratio against the calibration line. Sample runs included quality control specimens with high, medium, and low drug concentrations to ensure consistency of operating conditions.

Immediately prior to shipment to the UK, samples were retrieved from -80^o^C storage and 100 µl of plasma transferred to labelled cryovials for protein precipitation and *Mycobacterium tuberculosis* (*Mtb*) inactivation. 900 µl of precipitation / inactivation solution was added to each sample, vortexed, and left to incubate for 10 minutes. Biosafety experiments performed early in assay development showed that this method would eliminate live *Mtb* organisms in 100 µl samples of H37Rv broth, allowing removal from the biological safety cabinet and shipping as non-infectious Category B material. Extracted samples were sent on dry ice to Liverpool for bio-analysis.

In the Bioanalytical Facility, samples were vortexed for 10 seconds, centrifuged (4,000 rpm for 10 min), and 50 µl of supernatant transferred to a labelled 5 ml glass test tube. 20 µl of working internal standard solution was added to each tube with 1 ml of 0.1% formic acid in water. Samples were vortexed, 200 µl transferred to autosampler vials, and placed in the autosampler racks. Subject samples were analysed on a standard curve extracted in the similar way and quality control samples were interspersed in the batch to monitor the assay performance.

A typical assay started with the standard curve, followed by low concentration quality control sample, patient samples, medium concentration quality control sample, patient samples, and high concentration quality control sample. Plasma without internal standard was extracted with each batch and placed as blanks between samples at appropriate intervals. This demonstrated absence of contaminants in the matrix and minimised analyte carryover.

Samples were injected sequentially (2 µl) onto an AB Sciex 5500 system (Sciex). Chromatographic separation was achieved using a Phenomenex Synergi 80 Å polar end caped C18 150 x 2 mm 4µm column (Phenomenex), with mobile phase maintained at 300 µl/min (mobile phase A = 0.3% formic acid in water, mobile phase B = 0.3% formic acid in methanol). Quantification of ions resulting from fragmentation of parent compound was analysed by electrospray ionisation mass spectrometry with multiple reaction monitoring in positive ion mode. The ion source parameters used were turbo heater temperature of 550^o^C, curtain gas pressure 30 psi, ion source gas pressure of 50 (GS1) and 60 (GS2) psi, and a spray voltage of 5500 V. To minimise autosampler carry over, a rinsing solution mixture consisting of 50:20:30 MeOH:IPA:H_2_O, 35 µl/sec to a volume of 750 µl, occurred before and after every run. Data acquisition and processing was performed using Analyst and MultiQuant software (Sciex). Drug levels below the lower limit of quantification were omitted.

*Population pharmacokinetic modelling*

The plasma data were explored using the first-order conditional method of estimation with interaction using NONMEM^©^ (version 7.4.0, ICON Development Solutions). Interindividual variability was described using an exponential model; and for residual variability exponential, proportional, additive, or combined proportional and additive models were tested. In the absence of individual patient pharmacogenetic data the potential effect of acetylator status on isoniazid elimination was investigated using a mixture model for apparent clearance (CL/F).

Base model selection was achieved using likelihood ratio testing with the minimum objective function value (calculated using minus twice the log-likelihood of the data) as the criterion, and examination of relative standard error values and goodness-of-fit plots. A decrease in the objective function value of 3.84 or greater corresponded to a statistically significant difference between models (*p*=0.05, χ2 distribution, 1 degree of freedom).

Stepwise generalised additive modelling was then used to identify significant covariates for the model. An objective function value reduction of >3.84 was used as a cut-off for inclusion, and an objective function value change of >6.63 on stepwise deletion (corresponding to a significance level of 1%) as a prerequisite for retention in the final model.

Age, weight, and body mass index were tested as effects on both clearance (CL/F) and volume of distribution (V/F) all using covariate models of the form in Equation 1, describing the individual pharmacokinetic parameter estimate:

$$Equation 1: \theta_{x,i}=\theta_{x}*\left( \frac{{COV}_{i}}{{COV}_{mean}} \right)^{\theta_{COV}}*exp(\eta_{x,i})$$

where θ_x_ is the fixed effect, population estimate of the pharmacokinetic parameter CL/F or V/F, η_x,i_ the log inter-individual variability random effect component, drawn from a normal distribution with mean of 0 and variance ω_x_^2^, COV / COV_mean_ being the individual covariate value normalised to the dataset mean, and θ_cov_ being the exponent governing the covariate effect. Mean covariate values for normalisation were as follows: age: 34 years, weight: 51.1 kg, body mass index: 18.4 kg/m^2^.

Creatinine clearance, calculated from serum creatinine via the weight-normalised Cockcroft-Gault formula was modelled as a centred linear additive effect on clearance as follows:

$$Equation 2: \theta_{CL,i}=(\theta_{CL,"non-renal"}+\theta_{CL,"renal"}*\left( \frac{{CrCl}_{i}}{{CrCl}_{typical}} \right))*exp(\eta_{x,i})$$

The effects of sex and HIV status on CL/F and V/F were investigated as categorical effects using a covariate model of the form in Equation 3:

$$Equation 3: \theta_{x,i}=\theta_{x}*(\theta_{COV}^ COV)*exp(\eta_{x,i})$$

where COV is a dummy variable equal to 1 or 0 according to patient categorisation.

Two-thousand datasets were simulated using the parameter estimates defined by the final model to perform a visual predictive check in Perl-speaks-NONMEM (PsN, version 4.7.0).

Models describing epithelial lining fluid and alveolar cell exposures as ratios of plasma exposure were used to describe intrapulmonary pharmacokinetics. With only one intrapulmonary observation for pyrazinamide and ethambutol per participant, it was not possible to estimate both inter-individual in ratio parameter and residual variability in epithelial lining fluid and alveolar cell exposure in the analysis for these drugs. As estimates of residual error variance in lining fluid and alveolar cells for rifampicin and isoniazid were found to be similar, these values were fixed at 0.4 and 0.6 respectively for both pyrazinamide and ethambutol.

*Drug concentration determination*

The volume of epithelial lining fluid in bronchoalveolar lavage (V_ELF_) was derived from the following equation:

$$Equation 4: V_{ELF}=V_{BAL}\times(\frac{{urea}_{BAL}}{{urea}_{plasma}})$$

where V_BAL_ represents the total volume of lavage retrieved, and urea_BAL_ and urea_plasma_ the urea concentration in paired bronchoalveolar lavage supernatant and plasma samples respectively.

Epithelial lining fluid drug concentrations were calculated from the concentration in bronchoalveolar lavage supernatant using the following equation:

$$Equation 5: C_{ELF}=C_{BAL}\times(\frac{V_{BAL}}{V_{ELF}})$$

C_ELF_ and C_BAL_ represent the concentration in epithelial lining fluid and bronchoalveolar lavage supernatant respectively.

Drug concentrations in cell pellets were determined using the same 4 drug assay, except that intracellular standards and quality control samples were prepared in spiked samples of the human monocytic leukaemia cell line THP-1, at a cell count of 2 x 10^6^ cells. From the cell count obtained pre-centrifugation, liquid chromatography / tandem mass spectrometry output was converted into concentration of drug in ng/10^6^ cells (C_PELLET_). Given a mean macrophage cell volume of 2.42 µl/10^6^ cells [2], alveolar cell drug concentration (C_AC_) in µg/ml was estimated from:

$$Equation 6: C_{AC}=\frac{C_{pellet}}{2.42}$$

Individual-level post-hoc Bayesian estimates of plasma and intrapulmonary AUC, T_max_, and C_max_ were generated from the final models. AUC was calculated from simulated values of clearance (CL/F) using:

$$Equation 7: AUC=\frac{Dose}{(CL/F)}$$

where CL represents clearance at steady state in litres per hour; F is the oral bioavailability in the observation compartment (fixed to 1); and dose represents the dose in milligrams. T_max_ at steady state was calculated using the equation:

$$Equation 8: T_{max}= \ln(\frac{k_{a}}{k_{e}})/(k_{a}-k_{e})$$

where k_a_ represents the first-order absorption constant, k_e_ the first-order elimination rate constant, and ln the natural logarithm. C_max ­_was calculated from:

$$Equation 9: C_{max}=\left( \frac{k_{a}*Dose}{V*\left( k_{a}-k_{e} \right)} \right)*(\exp\left( -k_{e}*T_{max} \right)-\exp\left( -k_{a}*T_{max} \right))$$

For participants with 2 pharmacokinetic sampling visits, the mean AUC, C_max_ and T_max_ for rifampicin and isoniazid was recorded.

*Sputum bacteriology*

Baseline drug susceptibility of screening isolates was measured on custom-made microtitre plates (UKMYC3 Sensititre, Thermo Scientific). These assays used 96-well plates with increasing concentrations of rifampicin (0.015-16 µg/ml), isoniazid (0.015-16 µg/ml), and ethambutol (0.25-16 µg/ml). Pyrazinamide was not assessed due to its’ need for acidic test conditions. *Mycobacterium tuberculosis* isolates were revived on Löwenstein–Jensen slopes, colonies emulsified in saline and 0.2% Tween, and 100 µl of suspension at 0.5 McFarland standard inoculated into to 10 ml 7H9 broth with 10% OADC. 100 µl was transferred into each well of the microtitre plate, sealed, and placed in the incubator at 37^o^C. Plates were read when growth was clearly visible in the 3 positive control wells for each sample. The minimum inhibitory concentration (MIC) was recorded as the lowest concentration with no visible growth for each antibiotic.

**Supplementary Table 1**: Parameter value estimates for the final models

| **Parameter** | **Rifampicin** | | | **Isoniazid** | | | **Pyrazinamide** | | | **Ethambutol** | | |
| --- | --- | --- | --- | --- | --- | --- | --- | --- | --- | --- | --- | --- |
|  | **Typical value** | **RSE (%)** | **95% CI** | **Typical value** | **RSE (%)** | **95% CI** | **Typical value** | **RSE (%)** | **95% CI** | **Typical value** | **RSE (%)** | **95% CI** |
| Clearance (*CL/F*, L/h) | 12.2 | 9.5 | 9.9-14.5 | 13.7 | 5.5 | 12.2-15.2 | 3.8 | 3.8 | 3.6-4.1 | 43.0 | 4.2 | 39.4-46.5 |
| Volume of distribution (*V/F*, L) | 22.2 | 11.7 | 17.1-27.3 | 78.5 | 4.0 | 72.4-84.6 | 41.8 | 3.6 | 38.8-44.8 | 401.0 | 6.4 | 350.8-451.2 |
| First-order absorption constant (*K_a_,* h^-1^) | 0.24 | 3.5 | 0.22-0.26 | 3.29 | 20.4 | 1.97-4.61 | 1.09 | 12.1 | 0.83-1.35 | 0.36 | 9.3 | 0.30-0.43 |
| Epithelial lining fluid:plasma ratio  (*R_ELF/total-plasma_*) | 1.97 | 8.4 | 1.65-2.30 | 14.6 | 11.8 | 11.2-18.0 | 49.8 | 15.9 | 34.2-65.3 | 4.0 | 7.8 | 3.3-4.6 |
| Alveolar cells:plasma ratio (*R_AC/total-plasma_*) | 1.35 | 17.5 | 0.89-1.81 | 1.31 | 14.0 | 0.95-1.67 | 3.18 | 20.5 | 1.90-4.46 | 15.0 | 12.3 | 11.4-18.6 |
| **Covariates**  (Male) sex effect on clearance (*Θ_sex_*)  HIV effect on volume of distribution (*Θ_HIV_*)  Weight effect on volume of distribution (*Θ_weight_*)  Creatinine clearance effect on clearance (*Θ_CRCL_*) | 1.32  1.34  -  - | 9.3  15.2  -  - | 1.08-1.56  0.94-1.74  -  - | -  -  1.08  - | -  -  45  - | -  -  0.13-2.03  - | -  -  -  - | -  -  -  - | -  -  -  - | -  -  -  0.12 | -  -  -  36.4 | -  -  -  0.03-0.20 |
| **Interindividual variability**  Clearance (*ω_CL/F_*)  Volume of distribution (*ω_V/F_*)  Absorption constant (*ω_ka_*)  Epithelial lining fluid:plasma ratio (*ω_RELF_*)  Alveolar cells:plasma ratio *(ω_RAC_*)  Covariance *ω_CL/F_ ~ ω_V/F_* | 0.028  0.382  -  0.219  1.03  - | 55.6  29.3  -  27.4  27.8  - | -  -  -  -  -  - | 0.285  0.057  -  0.528  0.791  - | 10.8  29.2  -  22.7  26.7  - | -  -  -  -  -  - | 0.107  0.025  0.425  0.243  0.725  - | 20.6  73.5  24.7  75.7  51.0  - | -  -  -  -  -  - | 0.098  0.063  0.463  0.139  0.204  0.079 | 29.8  63.7  33.3  48.3  43.3  43.6 | -  -  -  -  -  - |
| **Interoccasion variability**  Clearance (*Κ_CL/F_*) | 0.051 | 49.8 | - | - | - | - | - | - | - | - | - | - |
| **Residual variability**  Plasma (*σ_plasma_*)  Epithelial lining fluid (*σ_ELF_*)  Alveolar cells (*σ_AC_*) | 0.575  0.396  0.650 | 3.8  11.2  7.6 | 0.532-0.618  0.309-0.483  0.554-0.746 | 0.418  0.436  0.557 | 3.9  10.3  9.5 | 0.386-0.450  0.348-0.524  0.453-0.661 | 0.239  0.4 FIX  0.6 FIX | 11.6  -  - | 0.185-0.293  -  - | 0.377  0.4 FIX  0.6 FIX | 5.9  -  - | 0.333-0.421  -  - |

Typical pharmacokinetic parameters for the population generated by the models. All four drugs were best described using one-compartment pharmacokinetic models, parameterised by clearance (CL/F), volume of distribution (V/F), and absorption rate constant. Epithelial lining fluid and alveolar cell exposures were described as ratios of plasma exposure (epithelial lining fluid:plasma ratio: *R_ELF/total-plasma_* and alveolar cells:plasma ratio: *R_AC/total-plasma_* respectively). Significate covariate relationships (from among age, weight, body mass index, sex, HIV status, and creatinine clearance) were identified using stepwise generalised additive modelling. Interindividual (between subject) variability describes the variation in pharmacokinetic parameters between participants. Interoccasion (within subject) variability was estimated for rifampicin and isoniazid as concentrations measured in intensive and continuation phases of TB treatment.

**Supplementary Figure Legends**

**Supplementary Figure 1: Intrapulmonary drug concentration and radiographic extent of right mid zone disease**

Intrapulmonary drug concentration versus percentage parenchymal involvement in the right middle zone. Baseline chest radiographs were scored by 2 independent readers according to a published method [3]. The coloured trend lines are loess lines. Green triangles and dashed lines: drug concentration in epithelial lining fluid; red circles and dotted lines: drug concentration in alveolar cells. RMZ: right midzone.

**Supplementary Figure 2**: **Visual predictive checks for the final plasma models**

Visual predictive checks for plasma rifampicin, isoniazid, pyrazinamide and ethambutol models, determined from 2,000 simulations, indicating satisfactory descriptions of the data. The lower, middle, and upper lines are the 5^th^ percentile, median, and 95^th^ percentile for the observed data. The shaded areas are the 95% confidence intervals for the 5^th^ percentile, median, and 95^th^ percentile for the simulated data. The open circles represent the superimposed observed concentrations.

**Supplementary Figure 3: Rifampicin plasma goodness-of-fit plots**

Observed concentrations (DV) versus population predictions (PRED, top left) and individual predictions (IPRED, top right), in which the solid line represents the line of identity. Absolute weighted predictions (WRES) versus population predictions (bottom left) and time after dose (bottom right).

**Supplementary Figure 4: Isoniazid plasma goodness-of-fit plots**

Observed concentrations (DV) versus population predictions (PRED, top left) and individual predictions (IPRED, top right), in which the solid line represents the line of identity. Absolute weighted predictions (WRES) versus population predictions (bottom left) and time after dose (bottom right).

**Supplementary Figure 5: Pyrazinamide plasma goodness-of-fit plots**

Observed concentrations (DV) versus population predictions (PRED, top left) and individual predictions (IPRED, top right), in which the solid line represents the line of identity. Absolute weighted predictions (WRES) versus population predictions (bottom left) and time after dose (bottom right).

**Supplementary Figure 6: Ethambutol plasma goodness-of-fit plots**

Observed concentrations (DV) versus population predictions (PRED, top left) and individual predictions (IPRED, top right), in which the solid line represents the line of identity. Absolute weighted predictions (WRES) versus population predictions (bottom left) and time after dose (bottom right).

**Supplementary Figures**

**Supplementary Figure 1: Intrapulmonary drug concentration and radiographic extent of right mid zone disease**


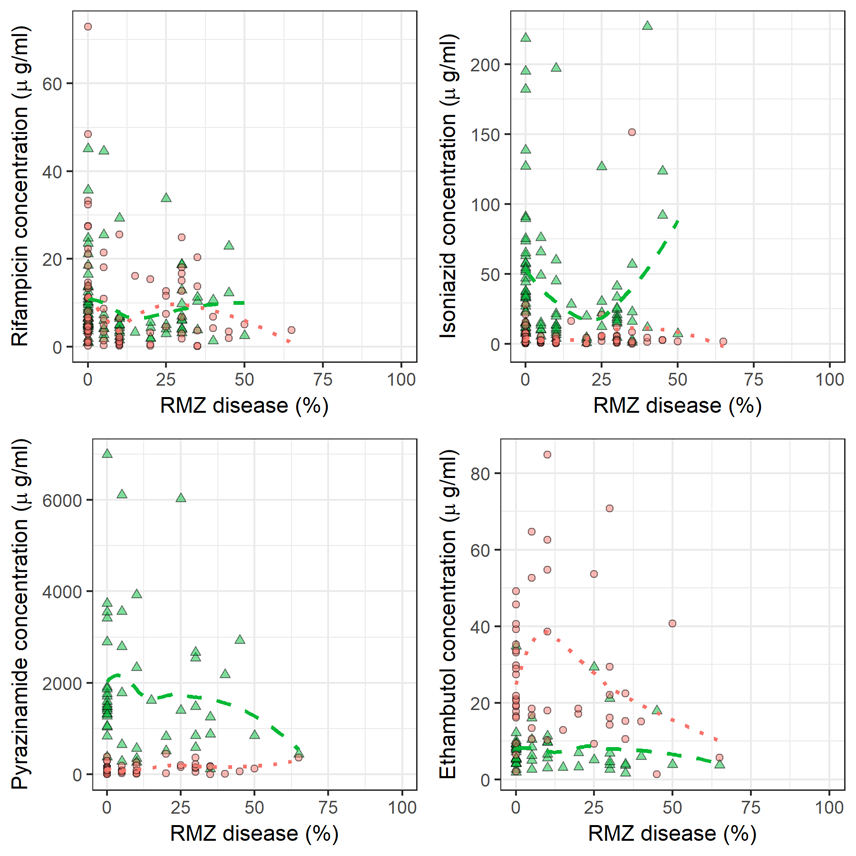


**Supplementary Figure 2**: **Visual predictive checks for the final plasma models**


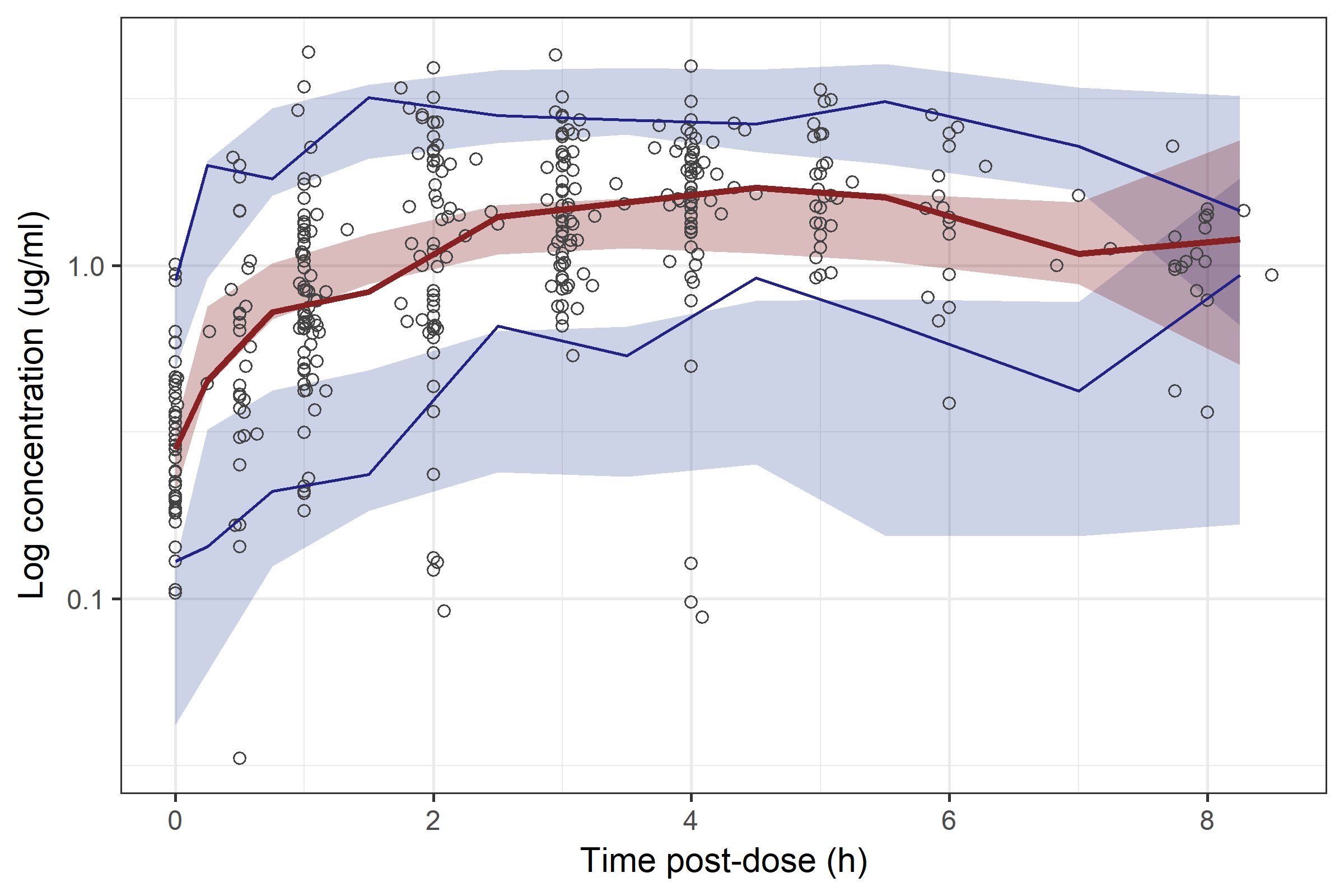

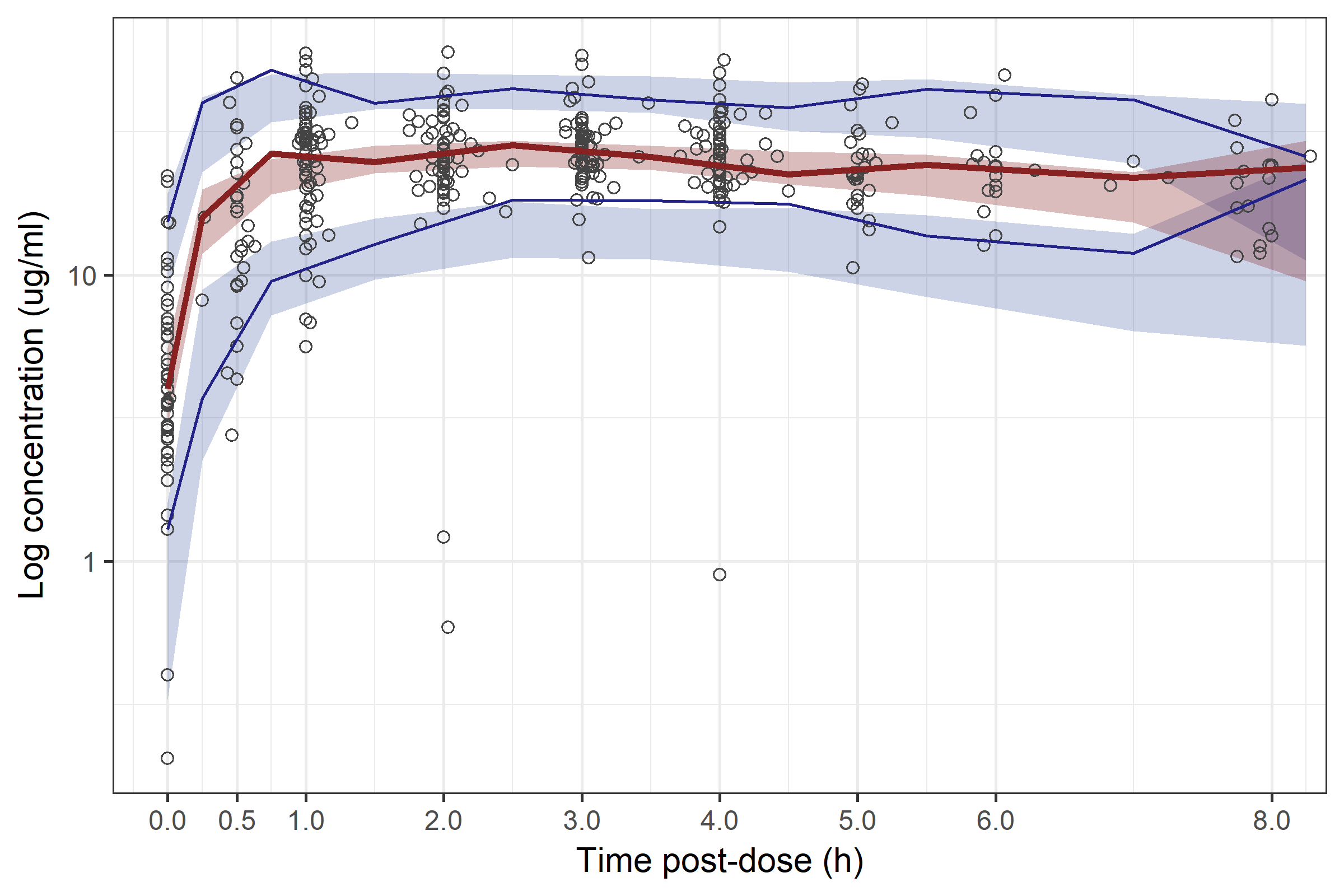

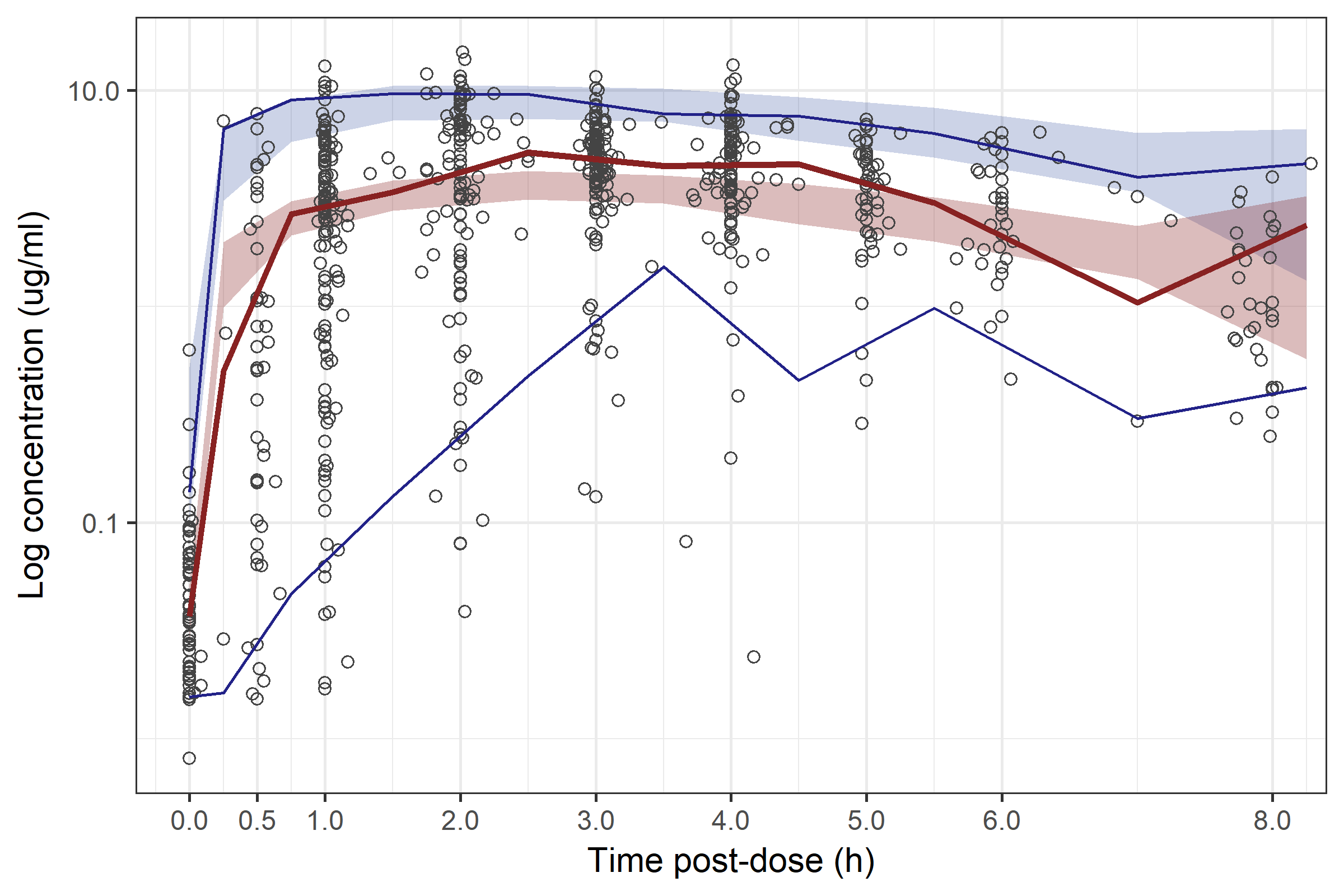

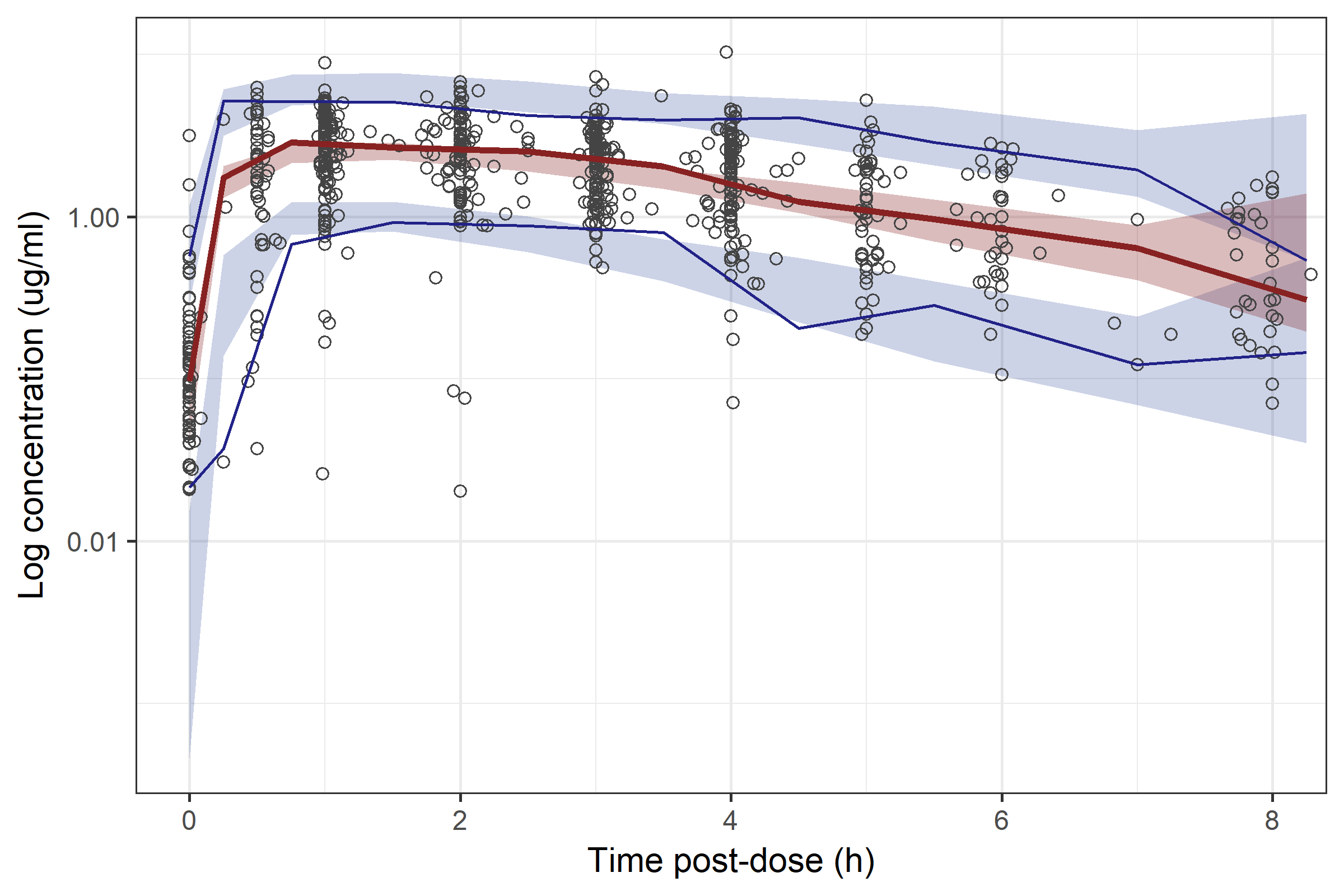


RIFAMPICIN

ISONIAZID

PYRAZINAMIDE

ETHAMBUTOL

**Supplementary Figure 3: Rifampicin plasma goodness-of-fit plots**

*
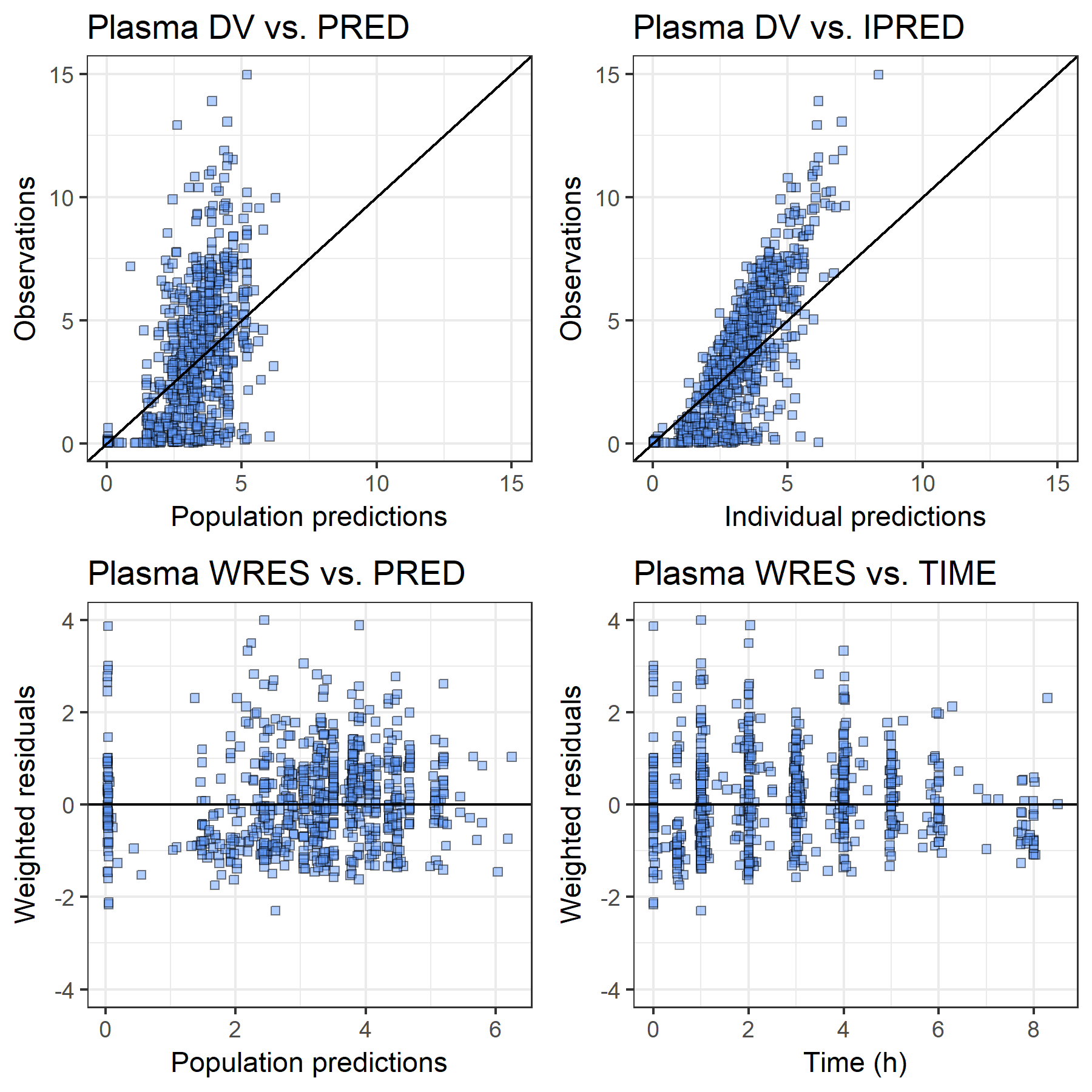
*

**Supplementary Figure 4: Isoniazid plasma goodness-of-fit plots**

*
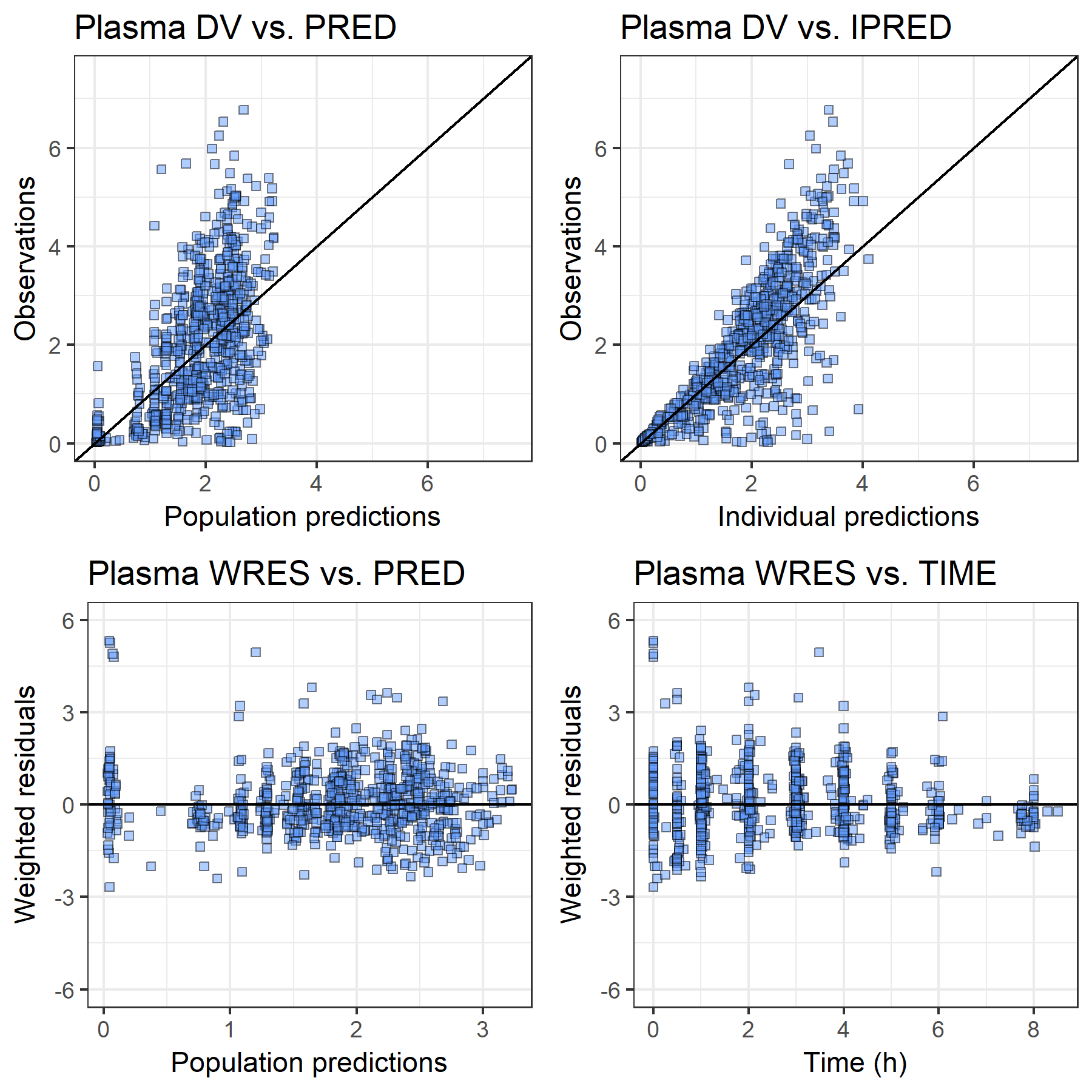
*

**Supplementary Figure 5: Pyrazinamide plasma goodness-of-fit plots**

*
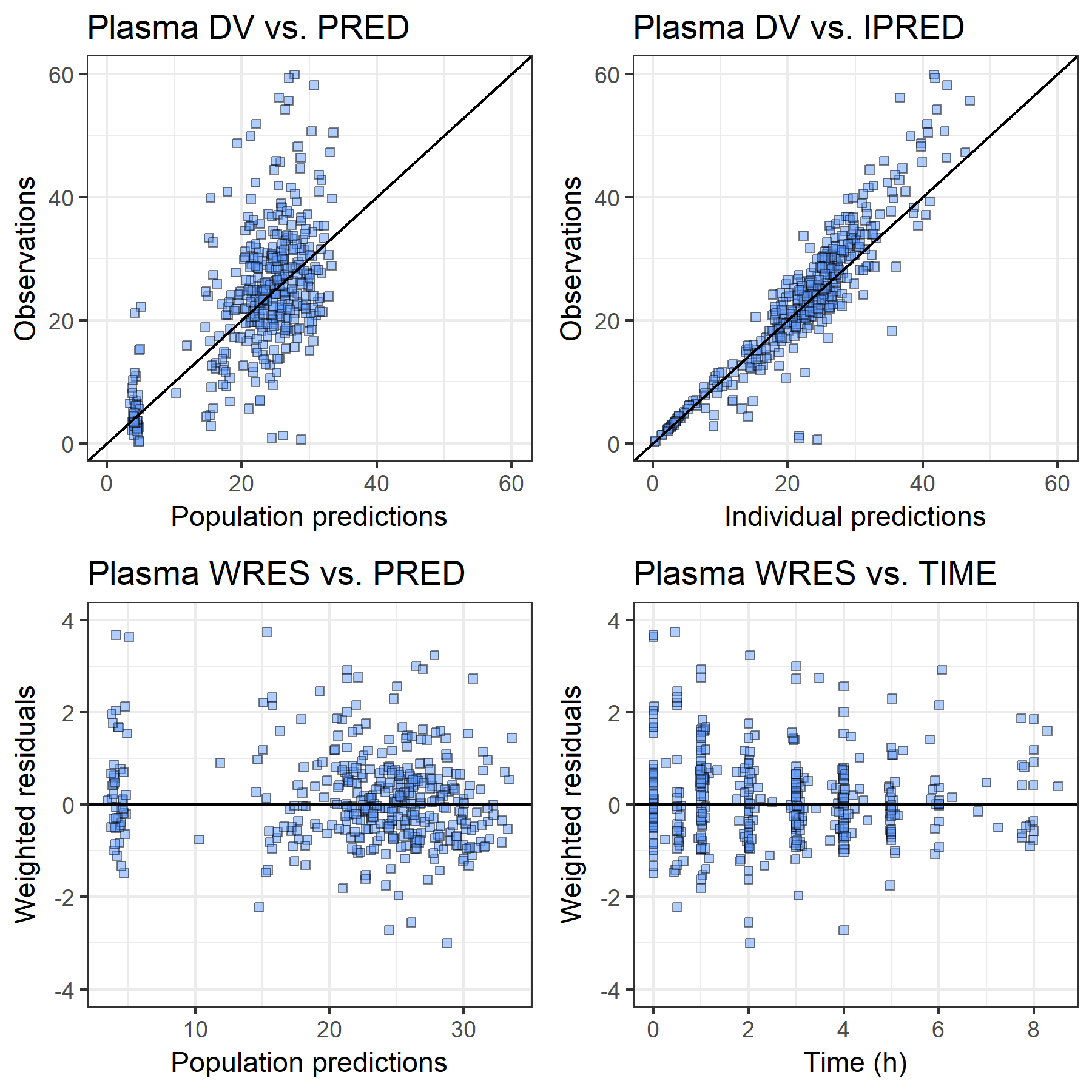
*

**Supplementary Figure 6: Ethambutol plasma goodness-of-fit plots**

*
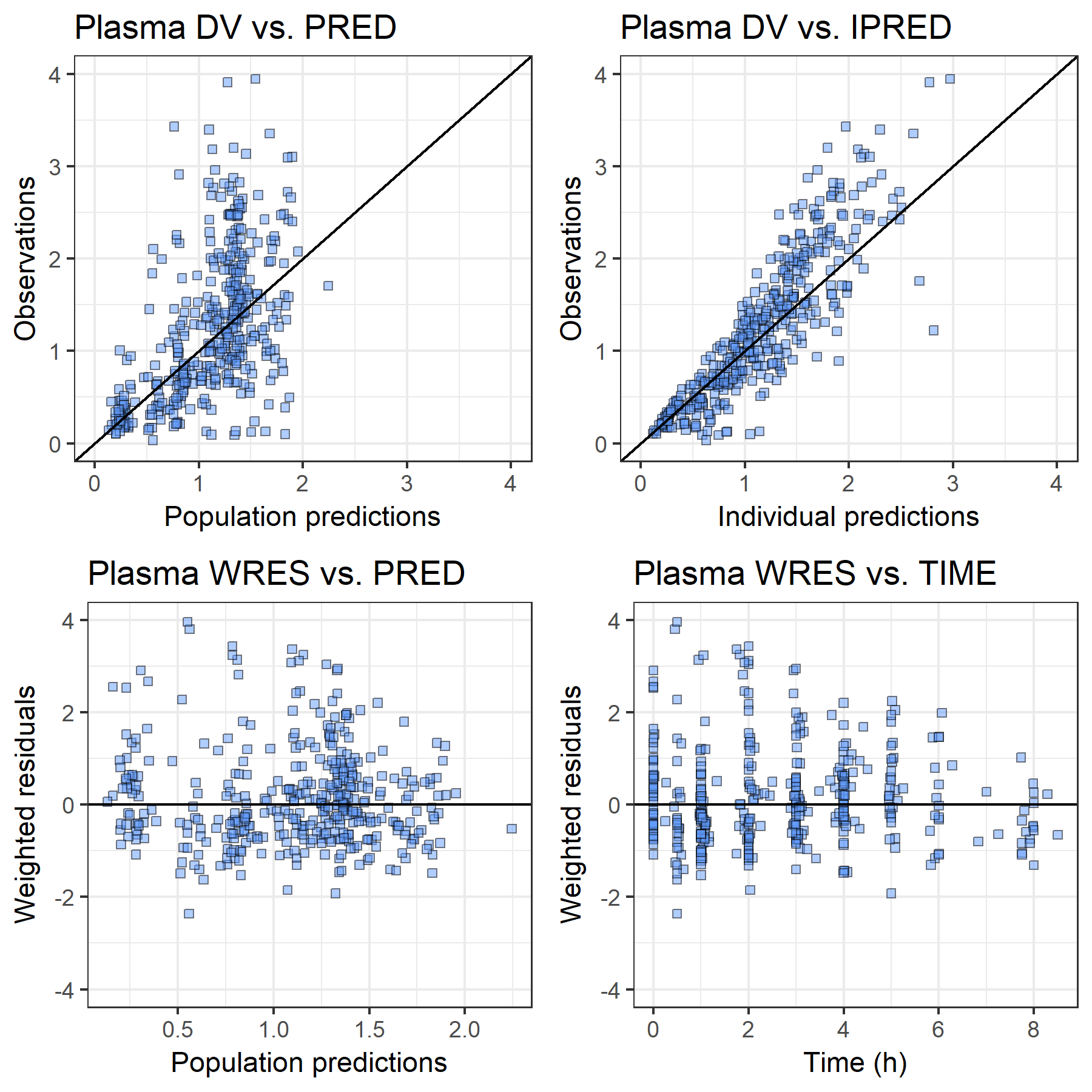
*

**Supplementary References**

1. Oken MM, Creech RH, Tormey DC, et al. Toxicity and response criteria of the Eastern Cooperative Oncology Group. Am J Clin Oncol **1982**; 5:649–655. Available at: https://www.ncbi.nlm.nih.gov/pubmed/7165009.

2. Baldwin DR, Wise R, Andrews JM, Ashby JP, Honeybourne D. Azithromycin concentrations at the sites of pulmonary infection. Eur Respir J **1990**; 3:886–890. Available at: https://www.ncbi.nlm.nih.gov/pubmed/1963411.

3. Ralph AP, Ardian M, Wiguna A, et al. A simple, valid, numerical score for grading chest X-ray severity in adult smear-positive pulmonary tuberculosis. Thorax **2010**; 65:863–869. Available at: https://www.ncbi.nlm.nih.gov/pubmed/20861290.
